# 256. Coinfection and Secondary Bacterial Infection in Hospitalized Patients with COVID 19 in a Fourth Reference Level Institution in Colombia: Incidence, Microbiology and Bacterial Resistance.

**DOI:** 10.1093/ofid/ofac492.334

**Published:** 2022-12-15

**Authors:** Patricia Reyes Pabon, Patricia Reyes Pabon, Natalia Cordoba Pulido, luisa Jimenez Alvarez, Claudia Sierra

**Affiliations:** clinica universitaria colombia, Bogota, Distrito Capital de Bogota, Colombia; clinica universitaria colombia, Bogota, Distrito Capital de Bogota, Colombia; clinica universitaria colombia, Bogota, Distrito Capital de Bogota, Colombia; clinica universitaria colombia, Bogota, Distrito Capital de Bogota, Colombia; clinica universitaria colombia, Bogota, Distrito Capital de Bogota, Colombia

## Abstract

**Background:**

The presence of coinfection and superinfection in hospitalized patients with COVID 19 varies, ranging between 3-14% in case of bacterial coinfection and 3 - 58% for superinfection. The objective of the study is to describe the incidence, type of infection and etiology in a cohort of patients with COVID 19 who required hospitalization.

**Methods:**

A retrospective series of adult patients with a confirmed diagnosis of COVID 19 who required hospitalization in the general ward or ICU and who presented coinfection or superinfection, between March and November 2020. The clinical and microbiological characteristics of patients who presented coinfection or superinfection are described

**Results:**

During the study period, 788 patients with COVID 19 who required hospitalization were evaluated. 6.2% presented coinfection, 49 coinfections were documented, 84% detected in patients who required admission to the ICU.

The coinfections detected were pneumonia 74%, tracheitis 11%, urinary tract infection 2% and soft tissue infection 2%. Gram negative bacilli were isolated in 58% of cases (*K. pneumoniae, H. influenzae, E. cloacae, E. coli*), 29% were gram positive (*S aureus. S pneumoniae, S. agalactiae*). Among gram negative bacilli, 12% showed resistance to 3 generation cephalosporins (3GCephR), no resistance to carbapenems (CR) was found.

Superinfection was detected in 18%, with 142 documented infectious events. 98% were hospitalized in the ICU with a mean hospitalization time of 9 days at the time of infection diagnosis.

The most frequent infections were tracheitis 49%, pneumonia associated with mechanical ventilation 21%, bloodstream infections 17%, pneumonia 8%, catheter-associated bacteremia 3.6%, urinary tract infection 0.7% and others 0.7%.

80% were gram negative (*K. pneumoniae, E. coli, E. cloacae, P. aeruginosa),* 16% gram positive and fungi 4% Among the isolated gram negative bacilli, 3% showed 3GCephR and 9% were CR.

**Table 1.**
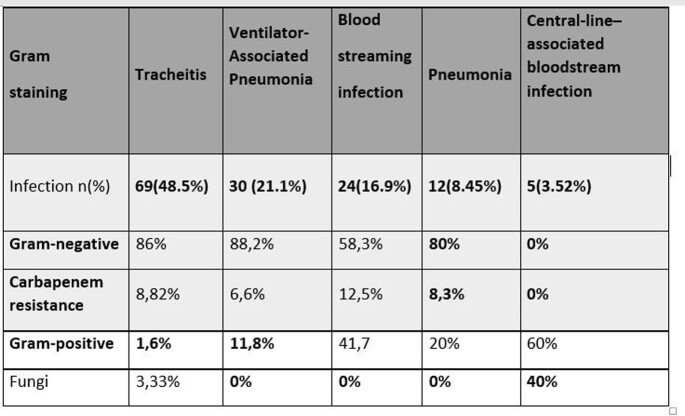
Types of superinfection and microbiological isolation

**Conclusion:**

The incidence of coinfection in patients with COVID 19 in this series is similar to that reported in the literature. Superinfection occurred in 18% of hospitalized patients, the majority hospitalized in the ICU. 78% of superinfections were from the respiratory tract. Gram-negative bacilli are the most frequently isolated germs in superinfection, with CR of the 9%.

**Disclosures:**

**All Authors**: No reported disclosures.

